# Acute pesticide poisoning amongst adolescent girls and women in northern Tanzania

**DOI:** 10.1186/s12889-020-8374-9

**Published:** 2020-03-06

**Authors:** Elikana Lekei, Aiwerasia V. Ngowi, Jones Kapeleka, Leslie London

**Affiliations:** 1 Tropical Pesticides Research Institute, P.O. Box 3024, Arusha, Tanzania; 2 School of Public Health and Social Sciences, Muhimbili University of Health and Allied Sciences (MUHAS), P.O. Box 65015, Dar es Salaam, Tanzania; 3 School of Public Health & Family Medicine, University of Cape Town, Anzio Road, Observatory, Cape Town, 7925 South Africa

**Keywords:** Acute pesticide poisoning, Adolescent girls & women, Northern Tanzania

## Abstract

**Background:**

Acute pesticide poisoning (APP) is reported to affect community health worldwide but its burden in Tanzania is unknown particularly in women. This study examines APP involving adult females and adolescent girls 10 to 19 years in 3 regions of Tanzania which are famous for coffee and vegetable production.

**Methods:**

Over the period of 12 months, health facility-based surveillance for cases of APP was implemented in 10 Tanzanian healthcare facilities in 2006.

**Results:**

The study identified 108 APP cases of whom 31 (28.7%) occurred amongst adolescent girls. Suicide was the leading poisoning circumstances (60.2%) and the most vulnerable women were 20–29 years old who comprised 38.4% of all cases with suicide as circumstance.

Organophosphates (OPs), zinc phosphide, paraquat and endosulfan were common amongst known reported poisoning agents. The annual APP incidence, mortality and Case Fatality Rate for women were 5.1/100,000, 0.2/100,000 and 3.7/100, respectively.

**Conclusion:**

APP amongst women in Tanzania is common and this call for diverse preventive interventions to reduce poisoning incidents.

## Introduction

Acute pesticide poisoning (APP) is documented as a serious public health problem in Tanzania [1, 2] and other parts of the world [3–9] due to its adverse human health effects. Evidence of APP occurring in women is documented in studies from Tanzania [2, 10], Uganda [11], Kenya [12], South Africa [13, 14], India [15], China [16], Nepal [17], Sri Lanka [18], Iran [19] and Ethiopia [20]. A 2002 review of APP globally suggested that pesticide-related morbidity and mortality is under-reported amongst women [21]. As a result, the Burden of Disease due to pesticide exposure, particularly amongst women, may be substantially underestimated. Similarly, amongst children, APP may be an under-reported problem globally. A WHO study reported that over 30% of the global burden of disease amongst children can be attributed to environmental factors, pesticides being a major contributor [22]. Childhood acute pesticide poisoning is documented in several countries including South Korea [23], South Africa [24], Canada [25], Turkey [26], and India [27]. Apart from accidental and occupational poisoning, adolescent risk-taking behavior can lead to attempted intentional pesticide self-poisoning. Reasons for adolescents’ intentional poisoning include emotional trauma from family stress or failed relationships with the opposite sex [28].

APP may also signal increased risk for long-term chronic health effects associated with pesticide exposure. For example, individuals surviving a single APP episode involving an OP are at risk of neuropsychiatric and neurological sequelae years after the episode [29, 30].

Age and gender are said to affect physiological susceptibility to the effects of exposure to toxic pesticides [31]. Biological factors, notably body size, and differences in physiological, hormonal, and enzyme functions between women and men, and between adults and children influence susceptibility to health hazards from exposure to pesticides [31]. With a generally higher proportion of body fat, women are more likely to store those pesticides that are fat-soluble in their tissues, which, if mobilized to become biologically active, can act as a continuous source of internal exposure linked to adverse chronic health outcomes [32]. Moreover, at particular stages of their lives, such as pregnancy, lactation, and menopause, women’s bodies undergo physiological changes that may also change their vulnerability to health effects from toxic chemicals [31].

Studies suggest that women’s exposure to pesticides in pregnancy is associated with miscarriages [33], premature births, birth defects, and low birth weight [31], largely because of trans placental exposure, as well as transfer via breast milk during post-partum breast-feeding [31].

Other health effects linked with pesticide exposure in women include pregnancy complications as reported in Tanzanian horticulture [2], depressed cholinesterase levels due to Organophosphate (OP) exposure as reported amongst South African female farm workers [34], and respiratory problems, including asthma, as reported in Brazil [35] and South Africa [34]. Hormonally-related cancers in females, including breast, thyroid, and ovary, were reported amongst women in North Carolina, USA [36]. Adolescent females are also at risk to the impacts of endocrine disrupting chemicals, and many pesticides have been linked to adverse impacts on sexual development amongst both boys and girls [37].

Exposure pathways for women are diverse. Women can be exposed to pesticides directly through poor pesticide handling in agriculture, ingestion involving deliberate swallowing of pesticides or their concentrates, inappropriate handling in pesticides retail shops and consumption of pesticide-contaminated food. Dermal absorption can occur with exposure to contaminated clothing, dust or residues on floors and other surfaces or objects. Other possible sources for exposure include pesticide drift and unattended empty pesticide containers [5]. While many pathways for women’s exposure may be similar to men’s, some pathways may be gender-specific. For example, women may also be exposed to pesticides through washing their husbands’ contaminated work clothes, or re-entering fields to conduct work earmarked for women following spray application [38].

Research on APP amongst women in Tanzania suggests that, firstly, agents reported to be responsible for poisoning include hazardous pesticides, particularly OPs used on food crops [2]. Secondly, although reports of occupational pesticide poisoning are uncommon in health facility records [1], studies have suggested that women may be at high risk for APP, given that women comprise of 80% of the agricultural labour force in Sub-Saharan Africa where pesticides are applied regularly [39].

Despite the prevailing reliance on pesticides for pest control [40], Tanzania currently has no surveillance system for APP. Poisoning is reported in the health management information system within Tanzanian hospitals with non-standardised information on the causative agent. Previous studies have reported the lack of specificity of reporting of pesticide poisoning in hospital information systems [10].

Because the magnitude of APP in adolescent and adult females in Tanzania is unknown, this study was undertaken with the aim of estimating the burden from APP and characterizing the patterns of APP amongst adolescent and adult females in Tanzania. Because Tanzania currently lacks a comprehensive surveillance system for acute pesticide poisoning, this study also could potentially generate recommendations to address this surveillance gap.

## Methods

### Data collection

This study was based on surveillance implemented at 10 health facilities in three regions of Tanzania (Mwanza, Kilimanjaro and Arusha) for APP admissions over a 12-month period from January 1st to December 31st 2006. These are regions where intensive vegetable and/or coffee production was associated with intensive pesticide use [10]. The 10 hospitals included regional hospitals, district hospitals, health centers and dispensaries. Medical data recorders were trained to use standardised methods to extract data on APP from facility records (both registers and patient folder) with regards to date of poisoning, location, gender, circumstances of poisoning and outcome. This paper focuses on the findings involving adolescent and adult females [10].

A case of APP in this study was defined as a diagnosis of APP made by the clinician and recorded in either the register or patient folder or both. In general, clinician diagnosis was based on a history of exposure (from the patient, relative, or accompanying person) to one or more pesticides and clinical manifestations of poisoning or specific laboratory test results compatible with APP, within 14 days of exposure. Cases were included in this study if they were females aged 10 years or older.

### Data analysis

APP cases captured were categorized by age using 10-year intervals (10–19, 20–29, 30–39 and 40+). Analysis of age further dichotomized age into two categories as (a) adolescent girls (10–19 years, as per WHO guidance on defining adolescence) and (b) mature Women (20 years or more). The variable age was used to evaluate the distribution of APP poisoning cases across various ages and age groups.

Poisoning circumstances were categorised as (a) suicide, (b) accidental, (c) occupational, (d) homicide, or (e) unknown. A second analysis of circumstances further dichotomized circumstances into two categories as (a) suicide versus (b) non-suicide. Categories were mutually exclusive. The variable circumstances of poisoning was used to evaluate actual reasons resulting into poisoning events.

The outcomes of APP were classified as (a) recovery, (b) absconded, (c) referred, (d) residual disability after discharge, (f) death, or (g) unknown and then dichotomized into (a) known versus (b) unknown. The variable outcome of poisoning was used to evaluate the end result of the poisoning in human health.

Pesticides responsible for APP were classified as (a) specific (meaning the active ingredient was identified), (b) nonspecific (meaning the active ingredient could not be specifically identified but its general category was known), or (c) unknown (active ingredient was not known to the clinician and/or not recorded and nor was there a general category associated with the poisoning). A second analysis further grouped the first two categories above into (a) Known versus (b) Unknown agents. The variable Agents responsible for poisoning was used to evaluate all specific products responsible for the poisoning events as well as most common chemical groups responsible for poisoning.

Comparisons involving circumstances of poisoning, agents, and age were conducted using chi-squared test and t-tests for continuous data such as age.

Bivariate analyses were conducted to explore associations involving agents as follows: (a) circumstances (Suicide/Non Suicide) and agents (Known / Unknown); (b) Age group (Adolescents / mature) and agents (Known / Unknown). Where specific information was known about the agent, analyses explored associations as follows: (c) Poisoning Agent (OP/ Non OP) and Age group (Adolescents / mature); and (d) Poisoning Agent (OP/ Non OP) and Circumstances (Suicide/ Non Suicide).

The strength of associations was estimated as prevalence risk ratios (PRR) with 95% confidence intervals. The analyses were conducted using SPSS statistical package version 16.0 and STATA statistical package version 10.0 [41, 42].

APP cases were used as numerator data to calculate morbidity rates stratified for geographical area, and age. To estimate denominators for rates, population census data were obtained from the Tanzania Bureau of Statistics based on a national census conducted in 2002 [43] and the population for 2007 adjusted for annual population growth of 2.7% [44]. The mortality rate was calculated as the number of deaths divided by the total population in the study area and then multiplied by 100,000 to yield death per 100,000 population. The Case Fatality Rate in this study was calculated by dividing the number of female deaths due to APP by the number of women diagnosed with APP and then multiplied by 100 to yield a percentage.

Because the study involved record review and no data were collected directly from individuals, no consent was required. To ensure confidentiality, patient names were replaced by codes, which were used as identifiers in data analysis. Ethical approval was secured from the Tropical Pesticides Research Institute (TPRI), the National Institute for Medical Research (NIMR) in Tanzania (Ref. NIMR/HQ/Vol XI/371), and the University of Cape Town (Ref. 328/2004).

## Results

Of the 10 health facilities followed up for 12 months during the study, 8 facilities reported 108 cases of APP amongst females 10 years and older. The 108 cases comprised 46.5% of the total of 230 cases (all male and female, adult and children) recorded in the course of 2006. Most cases (*n* = 77; 71.3%) were reported from the regional hospitals while others were reported from district hospitals, health centers and dispensaries. There were no instances of multiple poisonings in the same individual.

### Characteristics of cases reported

#### Age category of female APP cases

The age category with highest proportion of poisoned women was 20–29 years (44.4%) (Table 1).

**Table 1:** Age category and circumstances of poisoning for APP amongst females

Age category (Years)	Unknown	Accidental	Occupational	Suicide	Total
10–19	7	3	3	18	31 (28.7%)
20–29	11	7	5	25	48 (44.4%)
30–39	2	2	0	10	14 (13.0%)
40+	0	2	1	12	15 (13.9%)
Total	20 (18.5%)	14 (13.0%)	9 (8.3%)	65 (60.2%)	108 (100%)

Definitions: Suicide as poisoning arising from an act of deliberate self-harm; homicide as poisoning arising from a deliberate exposure experience by one person/s as a result of the actions of another person; occupational as poisoning arising amongst workers as a result of handing (mixing, spraying, storage or other usage) of pesticides for work purposes; accidental as poisoning occurring outside of the work context as a result of an unintended exposure; known as a circumstance that includes one or more of accidental, occupational, suicide or homicide; unknown as a poisoning case for which the circumstance was none of the above and was unknown

#### Circumstances of poisoning

The most commonly recorded circumstance of poisoning was attempted suicide (60.2%). Occupational APP was reported in 9 cases (8.3%) (Table 1). Three of the nine occupational cases of APP involved adolescent girls (10–19 years). Lack of data on circumstances of poisoning was highest in the age group 20–29 years (11 unknown cases).

There were 10 cases involving children aged 15 years or less. The largest proportion of cases arising from suicide attempts were found in adult females 20–29 years (38.4% of all suicide cases). Most cases involving accidents occurred amongst females between 10 and 29 years (10 cases).

Only four cases were reported to have resulted in fatalities (3.7%) involving victims who were aged 16, 17, 22 and 25 years old, and they all involved suicide. The outcome of APP was unknown for 27 females (25.2%). The majority of the unknown outcomes were concentrated in the age group 10–19 (11 cases) and 20–29 (12 cases).

#### Poisoning agents

The agents responsible for poisoning are reported in Table 2. Of the 108 cases in which pesticide poisoning occurred, in over half of the cases (60.2%), the exact pesticide involved was unknown. Where specific agents were reported (39.8%), organophosphates were most commonly named, accounting for 13 of the 21 cases where specific agents could be identified. All OPs reported were WHO Class II toxins with the exception of Chlorfenvinphos (3 cases), which is a WHO Class Ib toxin. Nonspecific products such as livestock dip (1.9%), food poisoning agents (food items contaminated with unknown pesticides) (12.0%), rat poison (5.5%) and insecticides (0.9%) accounted for 20.3% of all 108 products reported.

**Table 2:** Classification of agents responsible for poisoning in women in Tanzania

Product	Chemical group	WHO Class	Frequency	Percentage by category	Percentage all agents (*n* = 108)
1.Known products					
OP	OP	Ib and II	13	61.9	12.0
Sulphur	IN	IV	1	4.8	0.9
Endosulfan	OC	II	1	4.8	0.9
Zinc Phosphide	IN	Ib	4	19.0	3.7
Paraquat	BP	II	2	9.5	1.9
Sub total 1			**21**	**100.0**	**19.4**
2.Unspecific Products					
Livestock Dip	UN	UN	2	9.1	1.9
Food poisoning	UN	UN	13	59.1	12.0
Rat Poison	UN	UN	6	27.3	5.5
Insecticide poison	UN	UN	1	4.5	0.9
Sub total 2			**22**	**100.0**	**20.3**
3.Unknown					
UN	UN	UN	65	100.00%	60.2%
Sub total 3			**65**	**100.00%**	
All Agents			**108**		

*IN Inorganic, OP Organophosphate, OC Organochlorine*

*UN: Unknown BP* Bipyridylium derivative

The majority of the specific agents reported (95.21%) were WHO Class I and II pesticides and OPs (*n* = 13) accounted for 61.9% of all known poisoning agents (*n* = 21) (Table 2).

### Measure of associations

The proportion of circumstances due to suicide was higher in cases with unknown agents compared to cases with known agents and the difference was marginally significant (82.2% versus 65.1%; PRR unknown/known = 1.6; 95% CI = 0.9–2.9).

Adolescent girls were more likely to be involved in an APP where the agent was unknown than adult women (74.2% vs 54.5%, respectively; PRR Adolescent girls/Adult women = 1.9, 95% CI =0.9–3.9).

The proportion of cases with OP poisoning agents was significantly higher in:

- Adult women compared to Adolescent girls (PRR Adult women/ Adolescent girls = 1.3, 95% CI =1.01–1.6);
- Suicide compared to Non suicide circumstance (PRR Suicide/Non Suicide = 1.35, 95% CI – 1.1 – 1.6).

The proportion of suicide as a circumstance for APP was not significantly higher in adolescent girls compared to adult women (78.3% vs 73.4%, respectively; PRR adolescent girls/adult women = 1.1, 98% CI = 0.5–2.6).

### Acute pesticide poisoning rates

The annual Mortality rate (MR) and Case Fatality Rate (CFR) for women were 0.2 per 100,000 and 3.7 per 100, respectively.

The annual IR for APP was 5.1/100,000 with highest rates reported for the Arusha region. The age group 20–29 years reported the highest IR (8.3/100,000) (Table 3).

**Table 3:** Incidence rates for APP for women in Tanzania

	Poisoning cases per year	Population (2007)	Annual IR (Per 100,000)
Regions			
Arusha	36	495,525	7.3
Mwanza	57	1,072,533	5.3
Kilimanjaro	15	566,922	2.6
Total	**108**	**2,134,980**	**5.1**
Age groups			
10 to 19	31	748,449	4.1
20 to 29	48	577,650	8.3
30 to 39	14	333,549	4.2
40+	15	475,332	3.2
Total	**108**	**2,134,980**	**5.1**

## Discussion

In the period of 1 year this study identified 108 acute pesticide poisoning cases involving adolescent girls and adult women in 8 of the 10 facilities in this study. The fact that most facilities visited reported poisoning indicates that APP in women is widespread in the study area. Given that APP is under-reported in many developing countries [1, 13, 45], particularly amongst women [1], it is likely that there are further cases which are not captured due to the absence of an adequate surveillance system.

The most commonly recorded circumstance of poisoning in this study was suicide (60.2%). This proportion is lower than that reported among females in Sri Lanka (88.7%) [18] but higher than reported in Kenya (14.6%) [12]. The higher proportion of suicide in Sri Lanka women could be due to the Sri Lankan study including only rural facilities in areas of high pesticide use whereas this study included both rural and urban facilities (Additional file 1).

Occupational exposure as circumstance for APP was very low for females (only 8.3% of the cases reported). Again, this may be a function of under-reporting resulting from the absence of a comprehensive surveillance system for APP in Tanzania as well as misdiagnosis of signs and symptoms of pesticides exposure. Previous research suggests that occupational cases may be missed [1] since farmers do not present to hospitals, especially for less severe poisonings, but rather wait to recover or use alternative treatments [10].

The age distribution of APP in females suggests that the highest proportion of cases was found in the age group 20–29 years. This is broadly consistent with findings from studies in India [46, 47], South Africa [48] and Nepal [49] although the Nepalese and South African studies included females in the younger age range. Females aged 15 to 40 years may be involved in pesticide handling and may attempt suicide using pesticides as a result of easy access to toxic chemicals. Suicide risk in this age group is exacerbated by factors such as physical or sexual abuse, persistent psychosocial stresses, loss of social support, financial hardship, and heavy seasonal workloads. Similarly, depression arising from OP exposure [50, 51] may also contribute to increase risk for suicidal attempts.

Organophosphates (OPs) emerged as the single most important group of agents responsible for poisoning in females in this study. This pattern is consistent with studies conducted in India [47], Sri Lanka [18] and Nepal [49] and reflects the widespread use of organophosphate insecticides in Tanzania [52]. Previous research suggest that, broadly speaking, usage patterns appear to be correlated with APP in Tanzania [40].

Poisoning with OPs was significantly associated with older age (PRR Adult women/ Adolescent girls = 1.3, 95% CI =1.01–1.6). Adult women may be more likely to handle pesticides, both directly while working in the farm and indirectly through handling contaminated containers and clothing than adolescent girls and therefore have a higher chance of coming into contact with OP products. The lower frequency of handling pesticides in adolescent girls could also arise because of legislative measures to limit child labour and tasks associated with pesticide exposure of children [53].

The study also suggested an association between suicide and poisoning by OPs (PRR Suicide/Non Suicide = 1.35, 95% CI – 1.1 – 1.6). OP’s are freely available in Tanzania in various pesticide retail shops and can be easily used in suicide attempts. Controlling easy access to OPs may therefore be an important measure to prevent suicide. Investigating the causative factors in suicide attempts might also help to inform preventive strategies.

Other agents reported in APP cases included paraquat, Endosulfan and zinc phosphide, all of which are significant hazards. Endosulfan is associated with adverse male and female reproductive effects. Endosulfan metabolites bio-accumulate in adipose tissue, depending on their lipophilicity [54]. Endosulfan is said to be a neurotoxin, haematoxin, genotoxin, nephrotoxin and carcinogenic, causing both acute and chronic toxicity in males and females, with some laboratory evidence suggesting greater susceptibility amongst females [55]. As a chemical endocrine disruptor [56, 57] exposure to even small concentrations of Endosulfan can lead to serious health effects. Endosulfan has been banned in Tanzania for health risks since 2013 and it has also been earmarked for elimination under the Stockholm Convention on Persistent Organic Pollutants (POPs) and listed under the Rotterdam Convention on Prior Informed Consent [58].

Zinc phosphide is extremely toxic, causing acute respiratory distress in exposed persons due to the release of phosphine gas when it comes into contact with moisture [59]. However, it is not registered for use in Tanzania and its distribution is therefore illegal, its use being mainly for domestic rats control [1]. Because the main use of this highly hazardous product occurs within households, the proximity to human habitation is likely to increase risks for human poisoning. The illegal distribution also means that control of this hazard will require methods of enforcement different to products registered in Tanzania.

Paraquat is another pesticide commonly used as herbicide in Tanzania but also commonly misused for suicide attempts [1]. The concentrated product is highly toxic, has no antidote and, as a result, has been responsible for many fatal poisonings in Tanzania [1]. Poisoning of women using paraquat is also reported in Sri Lanka [18].

This study reported a relatively high proportion of APP cases (60.2%) with no data on the responsible agents, a proportion much higher than studies amongst women in Sri Lanka [18] and California [60]. This difference could result from the existence of better surveillance systems in Sri Lanka and California compared to Tanzania. For example, the study by Pearson and colleagues, which demonstrated how close collaboration between stakeholders in Sri Lanka made use of surveillance data to implement effective policies to prevent pesticide poisoning, confirms the presence of a robust surveillance system for APP in that country [61]. Absence of information on the poisoning agent was associated with younger age in that adolescent girls were more likely to be involved in an APP where the agent was unknown (PRR Adolescent girls/Adult women = 1.9, 95% CI =0.9–3.9). This higher proportion of unknown poisoning agents in adolescent girls could be linked to inexperience in pesticides handling in that they are less likely to identify the poisoning agents. Similarly, restriction of hazardous forms of child labour implies that young workers in agriculture will rarely handle pesticides. This may be another factor explaining why adolescents may be unable to identify poisoning agents.

Population-based APP incidence rates in this study (5.1/100,000) were far lower than reported in Nepalese females aged 15 to 50+ years (77.5/100,000) [47] and South Korean females aged 0 to 65+ years (27.4/100,000) [62]. This could be explained by (a) poorer hygiene resulting in unsafe pesticide handling practices in Nepal or (b) under-reporting due to the lack of a surveillance system in Tanzania. For the Korean study, there were also age differences in the study population including children aged 1 to 10.

Conversely, the incidence rates found in this study were about three times higher than reported in a US study [9] where better preventive practices and legislation in the US may explain this difference.

The APP cases found in this study may signal a problem of long-term adverse effects from pesticide exposures. Endosulfan is an example of an organochlorine insecticide which is fat soluble, may bio-accumulate in both environmental media and human tissues and has many adverse long-term effects. Those surviving APP may be left with residual neurologic impairment, particularly if the poisoning resulted in multi-organ failure or nervous system hypoxia. Research suggests that patients with a history of a single acute organophosphate or other pesticide poisoning are at risk of long-term neuropsychiatric sequelae [29]. Thus, while the absolute numbers of women and adolescent girls affected by acute poisoning is not that high, it may be the tip of an iceberg of wider exposures needing investigation.

To address the surveillance gap, hospital based surveillance data can be supplemented by promoting the community based self-reporting of APP whereby the community will capture mostly minor APP injuries. Similarly, forensic data source in Tanzania under the Ministry of Health can also be included to provide data on severe APP, particularly involving suicide and homicide cases. Tanzania lacks a comprehensive system such as the SENSOR-Pesticides surveillance program in the US which utilizes a very wide range of data sources and variables, such as poison center calls, workers’ compensation claims, and variables related to current job, crops worked, and pesticide handling status [8].

## Conclusion

The study indicates that acute pesticide poisoning is common among women in northern Tanzania with estimated IR of 5.1/100,000, MR of 0.2/100,000 and CFR of 3.7%. The most common known agents were WHO Class I and II pesticides including a number of Organophosphates. The most common circumstances for APP in women were suicide for women of the age group 10–29 years. To reduce suicide related to APP, attention should be paid to addressing the causes of suicide, safer storage, (storage far away from home premises), improved hygiene measures and control of access to toxic pesticides. Occupational APP and APP associated with child labour, though uncommon amongst hospital admissions, need to be addressed by improving safe handling conditions and notification systems, and stronger child labour regulation and enforcement. Multifaceted intervention efforts are needed to reduce acute pesticide poisoning among women in Tanzania.

## Supplementary information

**Additional file 1.** Comparison of different studies on Acute Pesticide Poisoning for their settings and findings [**References:**^8^, ^17^, ^18^, ^41^, ^62^–^67^].

## Data Availability

The detailed data can be made available on request from the first author (Principal Investigator).

## Abbreviations

APP: Acute pesticide poisoning
CI: Confidence interval
IR: Incidence rate
WHO: World health organization

## References

[1] Lekei EE, Ngowi AVF, London L (2016). Under-reporting of acute pesticide poisoning in Tanzania: Modelling results from two cross-sectional studies. Environ Health.

[2] Mrema JE, Ngowi AV, Kishinhi SS (2017). Pesticide exposure and health problems among female horticulture Workers in Tanzania. Environ Health Insights.

[3] Jensen HK, Konradsen F, Jørs E, et al. Pesticide use and self-reported symptoms of acute pesticide poisoning among aquatic farmers in Phnom Penh, Cambodia. J Toxicol. 2011; Article ID 639814. 10.1155/2011/639814. 10.1155/2011/639814 PMC3018643 21234245

[4] Konradsen F (2007). Acute pesticide poisoning a global public health problem. Dan Med Bull.

[5] Dalvie MA, Sosan MB, Africa A (2014). Environmental monitoring of pesticide residues from farms at a neighboring primary and pre-school in the Western Cape in South Africa. Sci Total Environ.

[6] Litchfield MH (2005). Estimates of acute pesticide poisoning in agricultural Workers in Less Developed Countries. Toxicol Rev.

[7] Calvert GM, Beckman J, Prado JB (2016). Acute Occupational Pesticide-Related Illness and Injury — United States. 2007–2011. MMWR Morb Mortal Wkly Rep.

[8] Centre for Diseases Control (2016). Acute Non occupational Pesticide-Related Illness and Injury — United States, 2007–2011. MMWR Morb Mortal Wkly Rep.

[9] Lekei EE, Ngowi AVF, London L (2014). Hospital-based surveillance for acute pesticide poisoning caused by neurotoxic and other pesticides in Tanzania. NeuroToxicol..

[10] Pedersen B, Ssemugabo C, Nabankema V (2017). Characteristics of pesticide poisoning in rural and urban settings in Uganda. Environ Health Insights.

[11] Bundotich JK, Gichuhi MM (2012). Acute poisoning in the Rift Valley provincial general hospital, Nakuru, Kenya. S Afr Fam Pract.

[12] London L, Bailie R (2001). Challenges for improving surveillance for pesticide poisoning: policy implications for developing countries. Int J Epidemiol.

[13] Ndlovu V, Jeebhay M, Dalvie M (2014). Asthma associated with pesticide exposure among women in rural Western cape of South Africa. Am J Ind Med.

[14] Mancini F, Van Bruggen AH, Jiggins JL (2005). Acute pesticide poisoning among female and male cotton growers in India. Int J Occup Environ Health.

[15] Zhang X, Zhao W, Jing R, et al. Work-related pesticide poisoning among farmers in two villages of southern China: a cross-sectional survey. BMC Public Health. 2011;429 10.1186/1471-2458-11-429. 10.1186/1471-2458-11-429 PMC3126745 21639910

[16] Lamsal D (2013). Acute pesticide poisoning: review of patients attending at emergency department in Chitwan Medical College. J Chitwan Med College.

[17] van der Hoek W, Konradsen F (2005). Risk factors for acute pesticide poisoning in Sri Lanka Tropical. Tropical Med Int Health.

[18] Shadnia S, Esmaily H, Sasanian G (2007). Pattern of acute poisoning in Tehran-Iran in 2003. Hum Exp Toxicol.

[19] Nigatu AW (2017). Respiratory Health and Acute pesticide intoxications among workers in the flower farm industry in Ethiopia. PhD Thesis.

[20] London L, de Grosbois S, Wesseling C (2002). Pesticide usage and health consequences for women in developing countries: out of sight, out of mind?. Int J Occup Environ Health.

[21] World Heath Organization (WHO) (2006). Principles for Evaluating Health Risks in Children Associated with Exposure to Chemicals.

[22] Lee WJ, Ko Y, Cha ES (2014). Acute pesticide poisoning among children in South Korea: findings from national health insurance claims data 2006-2009. J Trop Pediatr.

[23] Balme KH, Roberts JC, Glasstone M (2012). The changing trends of childhood poisoning at a tertiary children’s hospital in South Africa. S Afr Med J.

[24] Canwest News Service (06/2007) (2017). Pesticide Poisoning Bigger Problem than Canadians may think.

[25] Kalkan S, Erdogan A, Aygoren O (2003). Pesticide poisonings reported to the drug and poison information Center in Izmirm, Turkey. Vet Hum Toxicol.

[26] Gupta SK, Peshin SS, Srivastava A (2003). A study of childhood poisoning at National Poisons Information Centre, all India institute of medical sciences, New Delhi. J Occup Health.

[27] Eze JN, Ndu IK, Edelu BO (2018). Teenage organophosphate insecticide poisoning: an ugly trend in Enugu Nigeria. J Commun Med Primary Health Care.

[28] Savage EP, Keefe TJ, Mounce LM (1988). Chronic neurological sequelae of acute organophosphate pesticide poisoning. Arch Environ Health.

[29] Muñoz-Quezada MT, Lucero BA, Verónica Paz Iglesias VP, Muñoz MP (2016). Chronic exposure to organophosphate (OP) pesticides and neuropsychological functioning in farm workers: a review. Int J Occup Environ Health.

[30] UNDP, Environment & Energy Group (2011). Gender mainstreaming guidance series : chemicals and gender.

[31] La Merrill M, Emond C, Kim MJ (2013). Toxicological function of adipose tissue: focus on persistent organic pollutants. Environ Health Perspect.

[32] Naidoo S, London L, Burdorf A (2011). Spontaneous miscarriages and infant deaths among female farmers in rural South Africa. Scand J Work Environ Health.

[33] Dalvie A, Jeebhay MF, London L (2010). Health effects due to pesticide exposure among women in Western Cape.

[34] Faria NM, Facchini LA, Fassa AG (2005). Pesticides and respiratory symptoms among farmers. Rev Saude Publica.

[35] Lerro CC, Koutros S, Andreotti G (2015). Organophosphate insecticide use and cancer incidence among spouses of pesticide applicators in the Agricultural Health Study. Occup Environ Med.

[36] Park S, Chung CW (2018). Health behaviors related to endocrine-disrupting chemicals and the associated factors of adolescent Korean girls. Women Health.

[37] Fenske RA, Lu C, Negrete M (2013). Breaking the take home pesticide exposure pathway for agricultural families: workplace predictors of residential contamination. Am J Ind Med.

[38] United Nations Department of International Economics and Social Affairs (UNDIESA) (1991). The World’s Women: Trends and Statistics 1970–1990.

[39] Ngowi AVF, Mbise TJ, Ijani ASM (2007). Pesticides use by smallholder farmers in vegetable production in northern Tanzania. Crop Prot.

[40] Stata Corporation, STATA Version 10.0, statistics/data Analysis, 2007.

[41] SPSS (Statistical Package for Social Scientists) for Windows, Release 16.0 Standard Version, 2007.

[42] United republic of Tanzania, 2012 Population and Housing Census, March 2013.

[43] United Republic of Tanzania, Ministry of Planning, National Bureau of Statistics – Tanzania in Figures, 2010.

[44] Keifer M, McConnell R, Pacheco A (1996). Estimating under-reported pesticide poisoning in Nicaragua. Am J Ind Med.

[45] Somasundaram KV, Ashok PA, Shukla SK (2009). Epidemiological profile of OP poisoning cases treated at Pravara hospital, Loni, India. Indian J Prev Soc Med.

[46] Kumar MR, Kumar GP, Babu PR (2014). A retrospective analysis of acute organophosphorus poisoning cases admitted to the tertiary care teaching hospital in South India. Ann Afr Med.

[47] London L, Ehrlich RI, Rafudien S (1994). Notification of pesticide poisoning in the western Cape, 1987–1991. SAMJ..

[48] Gyenwali D, Vaidya A, Tiwari S (2017). Pesticide poisoning in Chitwan, Nepal: a descriptive epidemiological study. BMC Public Health.

[49] Carruth AK, Logan CA (2002). Depressive symptoms in farm women: effects of health status and farming lifestyle characteristics, behaviors and beliefs. J Community Health.

[50] London L, Flisher AJ, Wesseling C (2005). Suicide and exposure to organophosphate insecticides: cause or effect. Am J Ind Med.

[51] Lekei EE, Mkalanga HM, Mununa FT (2014). Characterization and potential health risks of pesticides registered and used in Tanzania. Afr Newsl Occup Health Safety.

[52] United Republic of Tanzania, “Elimination of child labour, Protection of Children and Young Persons,” in Law of the Child Act, 2009, (Act No. 21 of 2009) (Cap. 13), ISN TZA- 2009-L-86527).

[53] Cerrilloa I, Granadaa A, Lo’pez-Espinosa (2005). Endosulfan and its metabolites in fertile women, placenta, cord blood, and human milk. Environ Res.

[54] Amizadeh M, Saryazdi GA (2011). Effects of Endosulfan on human health. WebmedCentral Toxicol.

[55] Bretveld RW, Thomas CM, Scheepers PT (2006). Pesticide exposure: the hormonal function of the female reproductive system disrupted?. Reprod Biol Endocrinol.

[56] Andersen HR, Vinggaard AM, Rasmussen TH (2002). Effects of currently used pesticides in assays for estrogenicity, androgenicity, and aromatase activity in vitro. Toxicol Appl Pharmacol.

[57] Dubois M, Jansen K, Gupta A, Mason M (2014). Global Pesticide Governance by Disclosure: Prior Informed Consent and the Rotterdam Convention 107. Transparency in global environmental governance: critical perspectives.

[58] Yogendranathan N, Herath HM, Sivasundaram T (2017). A case report of zinc phosphide poisoning: complicated by acute renal failure and tubulo interstitial nephritis. BMC Pharmacol Toxicol.

[59] California Environmental Protection Agency Department of Pesticide Regulation. Summary of results from the California pesticide illness surveillance program 2014 (HS 1900). Sacramento; 2014.

[60] Pearson M, Zwi AB, Buckley NA (2015). Policymaking ‘under the radar’: a case study of pesticide regulation to prevent intentional poisoning in Sri Lanka. Health Policy Plan.

[61] Choi Y, Kim Y, Ko Y (2012). Economic burden of acute pesticide poisoning in South Korea. Trop Med Int J.

[62] Thundiyil JG, Stober J, Besbelli N (2008). Acute pesticide poisoning: a proposed classification tool. Bull World Health Organ.

[63] Razwiedani LL, Rautenbach PGD (2017). Epidemiology of organophosphate poisoning in the Tshwane District of South Africa. Environ Health Insights.

[64] Republic of South Africa (2003). National Health Act No. 61 of 2003.

[65] CDC (2005). Pesticide-related illness and injury surveillance: a how-to guide for state based programs.

[66] Ko S, Cha ES, Choi Y (2018). The Burden of Acute Pesticide Poisoning and Pesticide Regulation in Korea. J Korean Med Sci.

[67] Kim HJ, Cha ES, Ko Y (2012). Pesticide poisonings in South Korea: findings from the National Hospital Discharge Survey 2004–2006. Hum Exp Toxicol.

