# Supplementary material for: Acute pesticide poisoning amongst adolescent girls and women in northern Tanzania
Source: BMC Public Health. 2020 Mar 6;20:303. doi: 10.1186/s12889-020-8374-9 (PMC7065330; doi:10.1186/s12889-020-8374-9)
Supplement: Supplementary file 1 — Additional file 1. Comparison of different studies on Acute Pesticide Poisoning for their settings and findings [References:8, 17, 18, 41, 62–67]. [file 12889_2020_8374_MOESM1_ESM.docx]

**Additional file 1: Comparison of different studies on Acute Pesticide Poisoning for their settings and findings.**

| **Study first Author** | **Setting** | **Definition of APP** | **Age range of participants**  **(Years)** | **Incidence rates per 100,000** | **Age distribution of APPs (Years)** | **Proportion due to suicide/accident/occupational** | **Outcome** | **Agent** |
| --- | --- | --- | --- | --- | --- | --- | --- | --- |
| [Gyenwali](https://www.ncbi.nlm.nih.gov/pubmed/?term=Gyenwali%20D%5BAuthor%5D&cauthor=true&cauthor_uid=28673345) et al, 2017^48^ | Quantitative survey of pesticides poisoning among hospital-admitted cases in selected hospitals of Chitwan District of Nepal. | World Health  Organization’s (WHO) case  Definition.^62^ | 1 – 80 | **Adolescent girls:**  Age 15–19: (8.9)  **Adult females:**  Age 20–29: (49.9)  Age 30-49: 89.6  Age 50 and above: 29.5 | **Females:**  15-19: 22.0%  19 and older: 74.0% | **Females:**  Suicide – 90.8%  Accidental/Occupational- 9.2% | **Females&Males combined:**  Fatal: 3.8%  Unknown: 8.9% | **Females and Males combined**  Known: OP (39.6%),  PY (35.1%), Zinc phosphide (21.6%), Others (2%)  Unknown: 16.6% |
| [Van Der Hoek](https://onlinelibrary.wiley.com/action/doSearch?ContribAuthorStored=van+der+Hoek%2C+Wim) et al, 2005^17^ | Hospital review and characterization of patients with acute pesticide poisoning in a rural area of Sri Lanka | Any patient diagnosed by the attending physician as Pesticide poisoning, or treated as such. | 16 and older | **Females& males combined**: 163 | Age group (Males and females combined)  16 – 29 :62%  30 and above: 32% | **Females:**  Intentional (Suicide) : 88.7%  Non Intentional: 7.8%  Unknown : 3.3% | **Females&Males combined:**  Fatal:18% | **Females and Males combined**  **Agents:**  OP: 29.2%  Carbamate: 8.3%  Endosulfan: 5.8%  PY: 2.0%  Paraquat: 13.1%  Others: 9.6%  Unknown:29.7% |
| Lamsal , 2013^16^ | Retrospective Review of acute pesticide poisoning patients attending at emergency department in Chitwan medical college | Based on  history provided and pesticide container  Information if any. | 0 to 60 | Not given | **Females:**  10– 14: 11.3%  15 - 19: 43.3%  30 – 44: 37.7%  45 – 60: 7.5%  Over 60: 0% | Not given | **Females& Males combined:**  Fatal: 8.5% | OP: 43.3%  Endosulfan: 7.5%  Cypermethrin: 20.7%  Others: 28.5% |
| [Choi](https://onlinelibrary.wiley.com/action/doSearch?ContribAuthorStored=Choi%2C+Yeongchull) et al, 2012 ^61^ | Survey of APP cases from the Korean National Health Insurance Reimbursement database. | A case of pesticide poisoning reimbursed by National Health Insurance (NHI) or subsidized by National Medical Aid (NMA) | 0 – 65 | **Females**:  Age 0-19: 1.0  Age 20-64: 8.4  Age 65 and older: 28.3  **All** **females**: 9.2 | **Females:**  0-19: 2.5%  20-64: 59.25  65 and above: 38.3%. | Not given | **Mortality rate per 100 000 (Females):**  0-19: 0.0  20-64: 1.2  65 and above: 8.9  Overal 1.9 | Not given |
| Razwiedani et al, 2017^63^ | Cross-sectional study, review of retrospective, secondary data of OP poisoning cases over a 3-year period, reported at the Tshwane District surveillance office. | A case was based on notification requirements of the South Africa National Health Act No. 61 of 2003.^64^ | 1 to 40 | Not given | **Females:**  1-10 : 29.5%  11-20: 23.5%  21-30: 27.1%  31-40: 12.9%  40+: 11.0% | **Females:**  Accident: 23.5%  Suicide: 49.4%  Unknown: 27.1%  Occupational: Not given | **Death: Females** 5.9% | OP’s |
| Calvert et al, 2016 ^7^ | Collection of data on acute pesticide related illness and injury arising from no occupational exposure reported by 12 states in the US | Symptoms consistent with the pesticide  formulation involving one/more of:  (i) systemic signs/symptoms  (ii): dermatologic lesions, and (iii): Ocular lesions.^65^ | 1 and older | **Females (Occupational):**  Agricultural: 21.5  Non agricultural: 0.4  Industrial sector: 0.7 | Not given | **Females**:  **Non-Occupational:** 53.6% | **Death**: 0.1% (**males and females combined**) | **Major agents**:  OP’s,  Pyrethroids, Pyrethrins, Glyphosate, Carbamates, Triazine, Imidachloprid, Fipronil |
| Ko et al, 2018^66^ | (a) Data from Statistics Korea on mortality due to APP 2006–2014 (http://www.kostat.go.kr).  (b) Data on APP from Korean National Health Insurance claims  2006–2014. | prevalent case  defined as  episode  with ICD-10 code T60.0–T60.9 as primary  diagnosis.^67^ | 0 to 80 | **Females:** 5.9 | Age groups (**For both males and females combined in the year 2014**:  0-10: 0%  10-19: 0.3%  20-29: 1.4%  30-39: 6.3%  40-49: 15.5%  50-59: 25.3%  60-69: 20%  70-79: 22.3%  80 and above: 8.9% | **Males and females combined**  Suicide: 91%  Non Intentional: 8% | Death: **Females** 0.9% | Herbicide: paraquat  Non-specific  Others |
